# Screening, Diagnosis, and Investigation of Global Developmental Delay and Intellectual Developmental Disorder

**DOI:** 10.7759/cureus.99027

**Published:** 2025-12-12

**Authors:** Mariana Gouveia Lopes, Ana Carolina Alves, Ines Pedrosa, Caroline Lopes, Ana Lemos, Ester Pereira, Margarida Henriques

**Affiliations:** 1 Pediatrics Department, Unidade Local de Saúde da Região de Leiria, Leiria, PRT

**Keywords:** childhood, clinical protocol, diagnosis, etiological investigation, genetic origin, global developmental delay, intellectual developmental disorder, screening

## Abstract

Introduction: Global developmental delay (GDD) and intellectual developmental disorder (IDD) are significant neurodevelopmental conditions in the pediatric population. These conditions result from complex interactions between genetic and environmental factors.

Objectives: To standardize the screening, diagnosis, and etiological investigation of GDD/IDD in a Neurodevelopment outpatient setting and to determine the main etiologies and diagnostic yield of first-line tests.

Methods: A standardized protocol was established for the screening, diagnosis, and etiological investigation of GDD and IDD. The study population comprised children undergoing clinical evaluation for GDD or IDD, assessed prospectively between July 2018 and June 2021, with follow-up data collected until 2024. Developmental and cognitive assessment tools included the Griffiths Mental Development Scales - Third Edition (GMDS-III), the Wechsler Preschool and Primary Scale of Intelligence - Revised (WPPSI-R), and the Wechsler Intelligence Scale for Children - Third Edition (WISC-III). GDD was defined in children aged ≤5 years with a developmental quotient (DQ) or equivalent intelligence quotient (IQeq) below 70. IDD was defined in individuals aged >5 years with IQeq <70, or in cases of severe/profound IDD where standardized testing could not be administered. Statistical analysis was conducted using IBM SPSS® Statistics, version 29 (IBM Corp., Armonk, NY).

Results: A total of 123 children were included, comprising 34 with GDD and 89 with IDD, of whom 30 (34%) had a previous GDD diagnosis. The cohort was predominantly male (65%). In the GDD group, the Griffiths Scale was used in 19 children (mean DQ = 56) and the WPPSI-R in 15 (mean DQ = 51). In the IDD group, the Griffiths Scale was applied in eight (mean DQ = 66.5), the WPPSI-R in 5 (mean DQ = 66.5), and the WISC-III in 76 (mean DQ = 64). First-line etiological investigations included array-CGH in 52 (42%) children, identifying 8 pathogenic variants; FMR1 testing in 39 (32%), identifying two positive cases; and karyotype analysis in 13 (11%), identifying three abnormalities. Cranial MRI was performed in 22 (18%), with abnormal findings in 6 (27%), and EEG in 32 (26%), showing abnormalities in 18 (56%). Autism spectrum disorder was the most frequent associated diagnosis.

Conclusions: A structured protocol enhanced diagnostic consistency and efficiency in children with GDD and IDD. Genetic testing, particularly array-CGH, FMR1 analysis, and karyotype, proved most informative, yielding an overall etiological diagnosis rate of 11%. These findings highlight the importance of evidence-based protocols for comprehensive evaluation and genetic counseling.

## Introduction

The assessment of psychomotor development in early childhood is a fundamental component of pediatric care. However, there is considerable variability in the age at which typically developing children achieve developmental milestones.

Global developmental delay (GDD) is defined as a significant delay in two or more developmental domains, including gross and fine motor skills, speech and language, cognition, personal and social abilities, and activities of daily living. GDD may precede a diagnosis of intellectual developmental disorder (IDD), particularly when speech and language are affected, and the delay is moderate to severe [1-5]. However, developmental delays, especially mild ones, can be transient and, therefore, may not reliably predict IDD or other neurodevelopmental disorders [2,3,5]. IDD is characterized by significant limitations in both intellectual functioning and adaptive behavior, encompassing conceptual, social, and practical skills. Although manifestations may appear in early childhood, a formal diagnosis can only be made when standardized assessments are possible, typically from around five years of age [3,5,6]. The estimated prevalence of IDD is between 1% and 3%, similar to that of GDD [6].

Both GDD and IDD can be classified as syndromic or non-syndromic. When GDD or IDD are the only apparent clinical features, they are classified as non-syndromic. The presence of additional clinical features (such as dysmorphisms or congenital malformations) or comorbidities defines the syndromic forms [7,8]. Multiple etiological factors contribute to these neurodevelopmental disorders, often resulting from complex interactions between genetic predisposition, environmental influences, and developmental vulnerabilities [9]. An underlying cause can be identified in most individuals with moderate to severe GDD or IDD and in a significant proportion of those with mild forms. The overall diagnostic yield ranges from 24% to 80% [9,10].

Among cases in which an etiology can be identified (excluding environmental factors), up to 50% are attributable to genetic causes [9]. These include chromosomal abnormalities, pathogenic copy number variants, and single-gene disorders such as fragile X and Rett syndromes. Metabolic, mitochondrial, and imprinting disorders also contribute to the genetic spectrum associated with GDD and IDD [8,10,11].

Additionally, approximately 28% of cases are associated with central nervous system malformations or metabolic conditions [10]. However, purely metabolic etiologies are rare, accounting for only 0.25% to 0.42% of cases, although they remain clinically significant [11].

The diagnostic process for both GDD and IDD involves multiple sequential steps. After the initial screening, diagnostic confirmation must be obtained through objective and standardized developmental assessments. These evaluations should preferably be administered by professionals specialized in neurodevelopment [2,5,6].

According to the guidelines of the Individuals with Disabilities Education Improvement Act and the American Academy of Pediatrics, any child who screens positive for GDD or IDD should undergo standardized testing to assess intellectual functioning, along with a formal evaluation of adaptive behavior using validated assessment scales [9]. Several standardized instruments are available, each presenting specific advantages and limitations; however, there is currently no expert consensus regarding a single optimal tool for diagnostic evaluation [10].

A comprehensive medical evaluation should also be conducted concurrently. This includes a detailed medical history, covering personal and family background, physical examination, vision and hearing screening, neurological examination, and evaluation for behavioral and/or psychiatric disorders, as well as other systemic conditions [1,3,6,9]. A three-generation family history, along with a physical examination focused on general, neurological, dysmorphic, and dermatological abnormalities, represents the cornerstone of the diagnostic process. This approach not only aids in identifying the underlying condition or establishing a differential diagnosis, but also facilitates the identification of potential comorbidities that may require referral to the appropriate specialists [2,3,9,11]. If a known genetic condition is suspected, targeted genetic testing should be performed as a first-line investigation [11]. In the absence of a clear clinical suspicion, diagnostic workup may include broad genetic testing, assessment for associated malformations, blood tests, neuroimaging, electroencephalography, and sensory evaluations [12].

This study aimed to standardize the screening, diagnosis, and etiological workup of GDD and IDD through the development, implementation, and monitoring of a clinical protocol. Additional objectives included identifying the underlying etiologies and determining the diagnostic yield of first-line investigations. Secondary objectives were to determine the proportion of children with a positive GDD screening result without a confirmed diagnosis, and the proportion of children initially diagnosed with GDD who subsequently met criteria for IDD.

## Materials and methods

**Figure 1:**
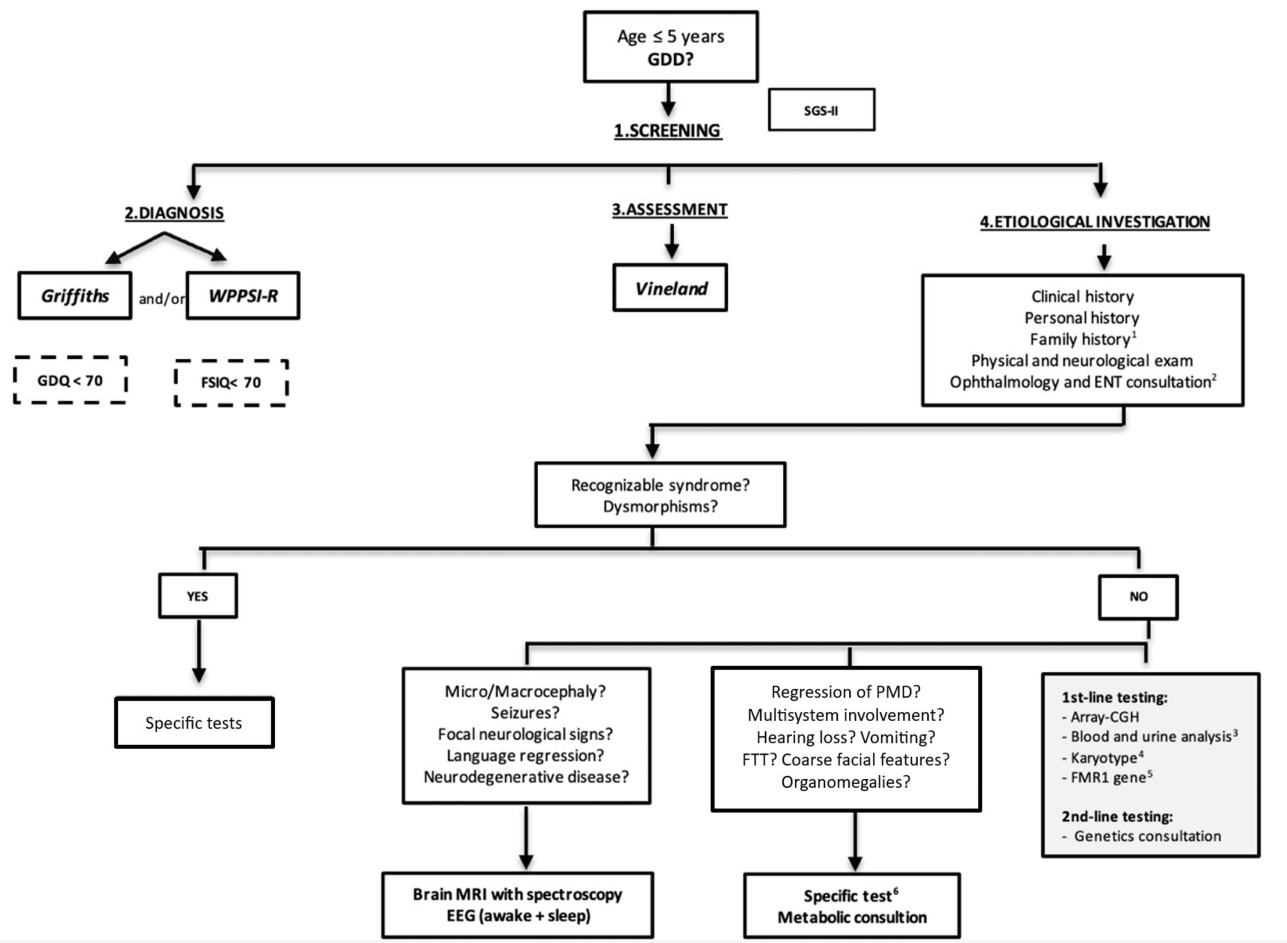
GDD algorithm EEG: Electroencephalogram; ENT: ear, nose and throat consultation; FSIQ: Full Scale Intelligence Quotient; FTT: failure to thrive; GDD: global developmental delay; GDQ: General Developmental Quotient; MRI: magnetic resonance imaging; PMD: psychomotor development; SGS-II: Schedule of Growing Skills II; WPPSI: Wechsler Preschool and Primary Scale of Intelligence – Revised. 1-If there is a family history of a molecular diagnosis consistent with the clinical presentation, the investigation should primarily target such etiology. 2-If there is suspicion of hearing or visual impairment, assessment should be guided by clinical manifestations and sensory screening (audiometric testing and pediatric ophthalmological screening according to the Portuguese Society of Ophthalmology Protocol)[13]. 3-Laboratory evaluation: complete blood count; glucose; urea; creatinine; sodium; potassium; phosphate; calcium; magnesium; UA: uric acid; CK: creatine kinase; AST: aspartate aminotransferase; ALT: alanine aminotransferase; TSH: thyroid-stimulating hormone; free T4: free thyroxine; venous blood gas analysis; ammonia; homocysteine; ceruloplasmin; and urinalysis (color, appearance/clarity, specific gravity, pH, glucose, proteins, ketones; bilirubin, urobilinogen, blood, nitrites, leukocytes / white blood cells. 4-If there is a family history of balanced chromosomal rearrangements or recurrent miscarriage/infertility. 5-Except in cases of multiple congenital anomalies, significant dysmorphisms, and/or microcephaly. 6-Based on clinical presentation.

**Figure 2:**
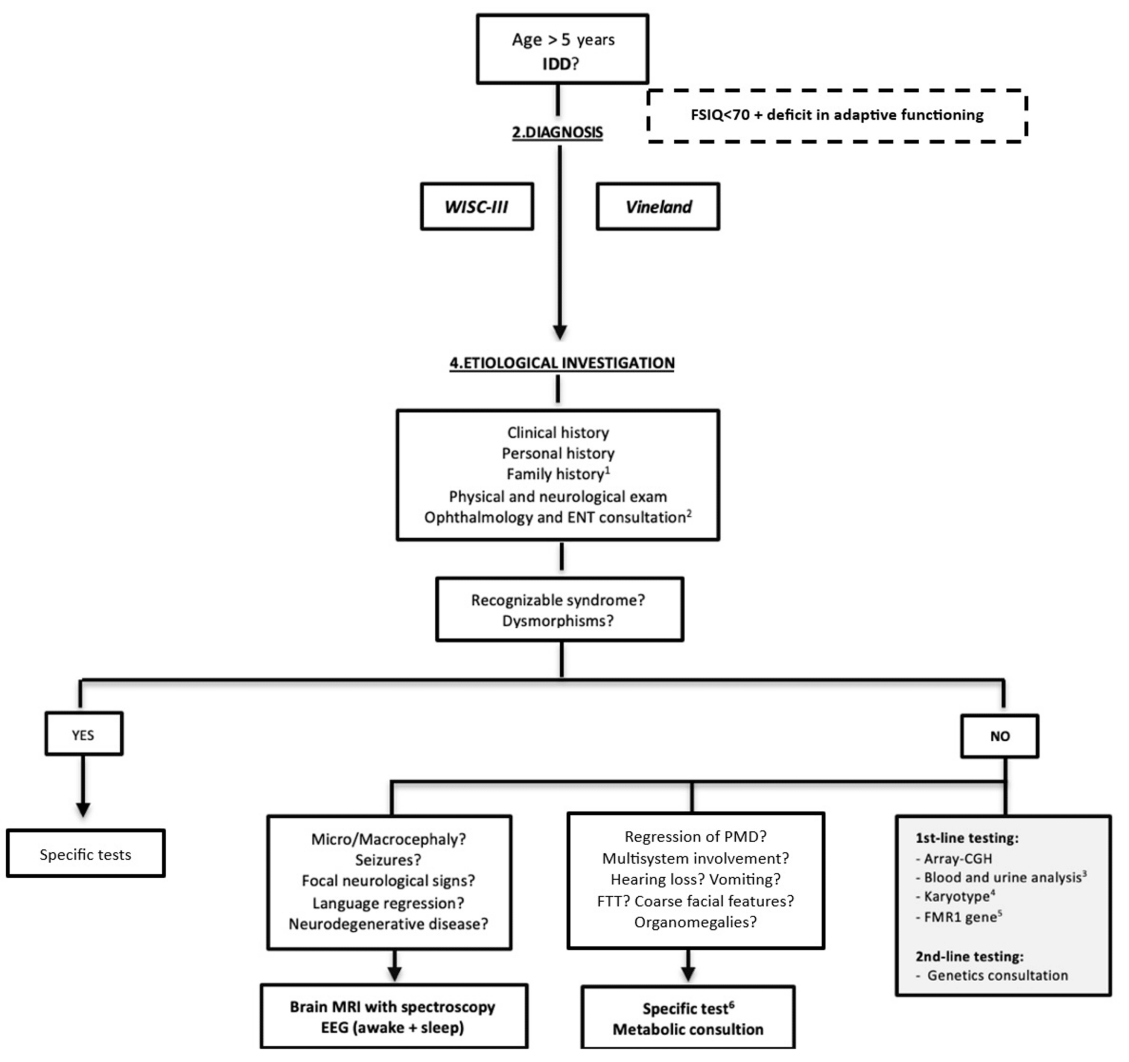
IDD algorithm EEG: Electroencephalogram; ENT: ear, nose and throat consultation; FSIQ: Full Scale Intelligence Quotient; FTT: failure to thrive; GDD: global developmental delay; GDQ: General Developmental Quotient; MRI: magnetic resonance imaging; PMD: psychomotor development; SGS-II: Schedule of Growing Skills II; WPPSI: Wechsler Preschool and Primary Scale of Intelligence – Revised. 1-If there is a family history of a molecular diagnosis consistent with the clinical presentation, the investigation should primarily target such etiology. 2-If there is suspicion of hearing or visual impairment, assessment should be guided by clinical manifestations and sensory screening (audiometric testing and pediatric ophthalmological screening according to the Portuguese Society of Ophthalmology Protocol) [13]. 3-Laboratory evaluation: complete blood count; glucose; urea; creatinine; sodium; potassium; phosphate; calcium; magnesium; UA: uric acid; CK: creatine kinase; AST: aspartate aminotransferase; ALT: alanine aminotransferase; TSH: thyroid-stimulating hormone; free T4: free thyroxine; venous blood gas analysis; ammonia; homocysteine; ceruloplasmin; and urinalysis (color, appearance/clarity, specific gravity, pH, glucose, proteins, ketones; bilirubin, urobilinogen, blood, nitrites, leukocytes / white blood cells. 4-If there is a family history of balanced chromosomal rearrangements or recurrent miscarriage/infertility. 5-Except in cases of multiple congenital anomalies, significant dysmorphisms, and/or microcephaly. 6-Based on clinical presentation.

This project was conducted in the pediatrics department of Hospital de Santo André, within the Local Health Unit of the Leiria Region. It was initiated with the development of a clinical protocol aimed at guiding the diagnostic process and etiological investigation of children referred to a neurodevelopmental outpatient clinic with suspected GDD or IDD. The protocol included two distinct diagnostic algorithms, one for GDD and another for IDD, designed to systematize and standardize the clinical approach. These algorithms are presented in Figures 1-2.

Definitions 

The definitions were based on the Diagnostic and Statistical Manual of Mental Disorders, Fifth Edition (DSM-5). GDD was defined in children up to five years of age as a delay of ≥ 2 standard deviations (SD) in two or more areas of psychomotor development on screening tests, or as a General Developmental Quotient (GDQ) or Full-Scale Intelligence Quotient (FSIQ) below 70 [14].

IDD was defined in children over five years of age as an FSIQ <70 along with an adaptive behavior deficit of ≥ 2 SD. For the purpose of the study, the authors adopted the DSM-IV classification of severity of IDD, according to FSIQ values: mild (50-69), moderate (35-49), severe (20-34), and profound (<20) [14].

Target population

The study included children aged ≤ 5 years with GDD, and children or adolescents > 5 years with IDD. Inclusion criteria consisted of positive screening for GDD up to 60 months of age and/or a GDQ/FSIQ <70 in children ≤ 5 years, and a GDQ/FSIQ <70 in children > 5 years for ID. The study included children who began their investigations between July 2018 and June 2021. Data collection continued until 2024. Cases with a previously established etiological diagnosis, such as Trisomy 21, Sotos-like syndrome, X-linked chromosomal abnormalities, and others, were excluded.

Assessment instruments

The evaluation of GDD included developmental screening using the Schedule of Growing Skills II (SGS-II) [15] in children aged 0-5 years, cognitive assessment with the Griffiths Mental Development Scales [16] in those aged 3-5 years, the Wechsler Preschool and Primary Scale of Intelligence (WPPSI) [17] in children aged 5-6 years, and functional assessment using the Vineland Adaptive Behavior Scales [18].

The assessment of IDD was performed using the Wechsler Intelligence Scale for Children - Third Edition (WISC-III) [19] for cognitive evaluation in individuals aged 6-17 years, complemented by functional assessment with the Vineland Adaptive Behavior Scales [18].

Etiological investigation

Etiological investigation for non-syndromic GDD or IDD was indicated when the clinical history and physical examination did not suggest a specific underlying condition. First-line assessment included laboratory testing (complete blood count, metabolic, hepatic, and hormonal panels, and blood gas analysis), urinalysis, karyotyping in cases with a suggestive family history (If there was a family history of balanced chromosomal rearrangements or of recurrent miscarriage/infertility), chromosomal microarray analysis (Affymetrix® CytoScan 750K, Thermo Fisher Scientific, Waltham, MA), and FMR1 gene testing by PCR (except in cases presenting with multiple congenital anomalies, dysmorphic features, or microcephaly).

Second-line evaluation was carried out during specialized genetics consultations at a tertiary pediatric hospital.

Statistical analysis

The variables analyzed included age at the start of follow-up, results of cognitive and functional assessment tests, presence of additional symptoms or associated diagnoses, findings on physical examination (including microcephaly or macrocephaly), family history, genetic and other investigations performed, and their respective results.

Categorical variables were described using frequencies and percentages, while continuous variables were presented as means and standard deviations or, in the case of non-normal distributions, as medians and interquartile ranges. Statistical analysis was performed using SPSS® software, version 29 (IBM Corp., Armonk, NY).

## Results

A total of 123 children and adolescents diagnosed with GDD or IDD were included in the study, of whom 80 (65%) were male. The median age at the first consultation was 54 months (IQR: 34.0-54.0; range: 1 month to 14 years and 10 months). Screening using the SGS-II was positive in 71 participants (58%), with a diagnosis of GDD confirmed in 59 cases, 28 of whom were subsequently reclassified into the IDD group.

Table 1 summarizes the characteristics of the GDD subgroup, which comprised only mild cases and showed a high rate of positive results on the SGS-II screening. Autism spectrum disorder was the most frequent comorbidity. The median age at the first evaluation was 38 months (IQR: 34-54).

**Table 1:** Characterization of the GDD Subgroup ASD: autism spectrum disorder; GDD: global developmental delay; SGS-II: Second Edition of the Schedule of Growing Skills

Variable	N	%
Severity (mild)	34	100
Sex (male)	23	68
SGS-II screening (positive)	31	91
Confirmed diagnosis, after negative SGS-II	3	9
Comorbidities (ASD)	11	32

The Griffiths Mental Development Scales were applied to 19 children (56%), and the WPPSI-R was applied to 15 children (44%). For functional assessment, the Vineland Adaptive Behavior Scales were applied to 29 children (86%), all of whom demonstrated deficits in adaptive behavior. Table 2 presents the results of the cognitive assessment.

**Table 2:** Cognitive Assessment of the GDD Subgroup GDQ: General Developmental Quotient; IQR: interquartile range; FSIQ: Full-Scale Intelligence Quotient; Mdn: Median; WPPSI-R: Wechsler Preschool and Primary Scale of Intelligence–Revised; GDD: global developmental delay

FSIQ/GDQ	Mdn	IQR
Griffiths Mental Development Scales, GDQ	56.0	31.0–68.0
WPPSI-R, FSIQ	51.0	44.0–57.0

Table 3 presents the characteristics of the IDD subgroup, which was predominantly composed of mild cases, with a prior history of GDD in approximately one-third of participants. The most common associated conditions were ASD. The median age at the first consultation was 75.5 months (IQR: 38.8-108.8).

**Table 3:** Characterization of the IDD Subgroup ASD: autism spectrum disorder; GDD: global developmental delay; IDD: intellectual developmental disorder

Variable	N	%
Severity (mild)	83	93
Sex (male)	50	56
Prior diagnosis of GDD	30	34
Comorbidities (ASD)	17	19

The Griffiths Mental Development Scales were administered to eight children (9%), the WPPSI-R was applied to five (6%), and the WISC-III was applied to 76 participants (85%).

For functional assessment, the Vineland Adaptive Behavior Scales were applied to 63 children (71%), with results consistent with deficits in adaptive behavior in 61 cases (97%). Table 4 presents the cognitive assessment results for the IDD subgroup.

**Table 4:** Cognitive Assessment of the IDD Subgroup FSIQ: Full-Scale Intelligence Quotient; GDQ: General Developmental Quotient; IQR: interquartile range; WPPSI-R: Wechsler Preschool and Primary Scale of Intelligence–Revised; WISC-III: Wechsler Intelligence Scale for Children – Third Edition; IDD: intellectual developmental disorder

FSIQ/GDQ	Mdn	IQR
Griffiths Mental Development Scales, GDQ	61.5	46.0–67
WPPSI-R, FSIQ	66.5	41.8–69.3
WISC-III, FSIQ	64.0	56.3–68.0

Cognitive and functional assessments revealed consistent deficits across both groups, confirming global impairments in developmental and adaptive functioning.

Etiological investigation

An etiological investigation was conducted in all 123 children and adolescents diagnosed with GDD or IDD. Analytical testing was performed in 85 participants (69%). No evidence of multisystem involvement was identified in any of the patients.

Genetic study

Genetic testing was performed in 105 participants (85%) to investigate potential underlying causes of developmental disorders. Array comparative genomic hybridization (array-CGH) was conducted in 52 cases (42%), revealing 24 genomic alterations in 19 patients, 8 of which were classified as pathogenic (diagnostic yield: 15%). Karyotyping was performed in 13 cases (11%), identifying three abnormal results consistent with known chromosomal syndromes (diagnostic yield: 23%, in selected cases). FMR1 gene analysis was conducted in 39 children and adolescents (32%), identifying two cases consistent with Fragile X syndrome (FXS) (diagnostic yield: 5%, in selected cases).

Additionally, targeted mutation analysis of the SALL1 gene identified 1 case of Townes-Brocks syndrome based on clinical suspicion. Overall, a genetic etiology was established in 11% of GDD/IDD cases. The distribution of tests performed, alterations detected, pathogenic variants, and corresponding diagnostic yields is summarized in Table 5.

**Table 5:** Genetic testing results and diagnostic yield Array-CGH: array-based comparative genomic hybridization; FMR1: fragile X mental retardation 1.

Test (n, %)	Alterations detected (n)	Pathogenic Alterations (n)	Diagnostic yield (%)	Alteration
Array-CGH (n=52, 42%)	24	8	15%	1q21.1 microduplication syndrome; 7p14.1 deletion syndrome; 17p12 duplication syndrome; 15q11.2 interstitial deletion; chromosome 12 duplication; 3p25.2p24.3 interstitial deletion; 10q26.2q26.3 interstitial deletion; 16p12.2 interstitial deletion
Karyotype (n=13, 11%)	3	3	23%	47, XXY (Klinefelter syndrome); 45, X (Turner syndrome); Chromosome 3 structural rearrangement
FMR1 gene analysis (n=39, 32%)	2	2	5%	Fragile X syndrome

Additional investigations and referrals 

Specific metabolic tests were performed in 16 children and adolescents (13%), with abnormalities detected in three cases. These included alterations in plasma amino acid levels, urinary orotic acid, and plasma homocysteine, all of which were considered to be of uncertain clinical significance.

Brain magnetic resonance imaging was performed in 22 participants (18%), revealing abnormalities in six cases (27%). Two findings were consistent with sequelae of neonatal stroke, while the remaining abnormalities were nonspecific. Electroencephalography was conducted in 32 cases (26%), with abnormalities observed in 18 (56%). All findings were nonspecific and did not contribute to etiological clarification. A total of 41 children (33%) were referred for medical genetic consultation, and three (2%) were referred for metabolic disorder consultation, corresponding to those with abnormal results in specific metabolic testing.

## Discussion

The use of clinical protocols is an essential tool for standardizing and systematizing the approach to specific health conditions. Clinical algorithms, as graphical representations of a logical sequence of diagnostic and therapeutic decisions, help to harmonize procedures and improve the efficiency of medical evaluations [20]. In the present study, the development and implementation of a specific protocol proved useful in standardizing clinical management.

The likelihood of identifying an underlying etiology in cases of GDD or IDD varies according to the methodology used and the severity of the clinical presentation. In cases of severe IDD, an underlying cause can be identified in up to 80% of cases, whereas in mild IDD, this percentage is significantly lower, approximately 24% [6]. Although the etiology remains unknown in many cases, it is estimated that genetic factors contribute to approximately 50% of all cases [8,21]. This proportion appears to increase with the severity of the condition, reaching up to 60% [8]. In this context, genetic investigation has become a key component of diagnostic evaluation [22].

In this study, a genetic etiology was identified in 11% of GDD/IDD cases. Although the overall diagnostic yield appears lower than that reported in some previous studies, this finding is unlikely to be due to limited testing, as genetic investigations were performed in 85% of participants. Rather, the relatively modest yield may reflect the heterogeneity of GDD/IDD, the limitations of current genetic testing approaches, and the predominance of milder phenotypes in the study population.

Among the children diagnosed with GDD/IDD, there was a predominance of males, which is consistent with what has been described in the literature [7,8]. This finding may, in part, be explained by the higher frequency of X-linked genetic disorders [8]. It is estimated that mutations in these genes may account for up to 10% of GDD/IDD cases [5,9]. 

Among the 71 children who screened positive for GDD using the SGS-II, 59 received a confirmed diagnosis, while 12 did not. According to the literature, several factors are known to influence child development, including prematurity, nutritional deficiencies, chronic illnesses, prolonged hospitalizations, and family neglect, which are often associated with transient forms of GDD [8,12]. In our study, these factors were not identified, suggesting that the findings may reflect a predominance of milder phenotypes. 

A diagnosis of IDD was confirmed in 89 participants, of whom only 30 had a prior diagnosis of GDD. Although previous studies indicate that many children with GDD later meet criteria for IDD, supporting the notion of a developmental continuum between these conditions [2,6], this pattern was not clearly observed in our cohort, possibly because most IDD cases were of milder severity.

The heterogeneous clinical presentation of IDD contributes to delayed diagnosis, as children with milder forms may initially exhibit apparently typical motor, language, and cognitive development, thereby hindering early identification [7,9]. Mild forms of IDD are often not recognized until between five and nine years of age [7]. Such diagnostic delays may partly reflect parental reluctance to acknowledge the condition, as well as concerns about its potential psychosocial consequences for the child, including fear of social rejection. In contrast, children with more severe forms are typically identified before two years of age [8].

Neurological comorbidities, including epilepsy, ADHD, and ASD, are frequently reported in children with GDD and IDD [22], with ASD emerging as the most prevalent comorbidity in the present study. In a cohort of children with GDD aged 24-60 months, ASD was identified in 62.3% of cases, reinforcing the concept that ASD represents one of the most common comorbidities in this population [23]. In our study, the proportion of children with GDD and comorbid ASD was 32%. With regard to ASD in individuals with IDD, prevalence estimates vary considerably, ranging from approximately 4% to 33%. Systematic reviews indicate that at least 10% of individuals with ID present with ASD [24], while other studies have reported prevalence rates as high as 30%, depending on sample characteristics and diagnostic methodologies [23]. In our study, the diagnosis of autism was primarily clinical, based on developmental history, direct observation of behavior, and the presence of symptoms from early development with functional impact. Standardized instruments, such as the ADOS, were used to support the diagnostic process by providing a structured assessment of social and communicative behaviors. The proportion of children with IDD and comorbid ASD in our cohort was 19%.

Regarding the effectiveness of first-line investigations, array-CGH is considered the first-line diagnostic test for the etiological investigation of GDD/IDD, following a detailed clinical history and physical examination [1,7-9,11,12]. Its diagnostic yield ranges from 10% to 20%, with higher efficacy in moderate to severe cases (20-30%) compared to mild cases (12-19%) [7,10,12]. In the present study, the diagnostic yield of array-CGH was 15%, consistent with the literature.

Karyotyping is recommended when there is clinical suspicion of aneuploidy (e.g., trisomy 21, Turner syndrome), a suggestive family history of chromosomal abnormalities, or recurrent miscarriages. In the latter case, many authors suggest initial parental karyotyping [2,6]. However, karyotyping is not currently considered a first-line test in most cases, as array-CGH provides at least twice the diagnostic yield [2,12]. Although the diagnostic yield of karyotyping is generally reported to be between 5% and 10%, our study demonstrated a substantially higher yield of 23%. This discrepancy is likely explained by the selective use of karyotyping in our cohort. In contrast to studies in which the test is applied broadly to all children with GDD/IDD [7,12], karyotyping in our study was reserved for cases with specific clinical indications, such as suspected aneuploidy or a family history suggestive of chromosomal abnormalities.

FXS is among the most prevalent monogenic causes of GDD and IDD, with an estimated prevalence of 1.4 per 10,000 in boys and 0.9 per 10,000 in girls [11]. Although this condition is frequently associated with distinctive craniofacial features, such as an elongated or narrow face, high forehead, prominent ears, prognathism, macroorchidism, and mild macrocephaly, these characteristics may be absent or subtle during early childhood [8,11].

Current guidelines from the American College of Medical Genetics and Genomics and the American Academy of Pediatrics recommend that FMR1 testing be included as part of the first-line diagnostic investigations for children with GDD or IDD, particularly when there is a family history suggestive of FXS [6] or other FMR1-related neurodevelopmental conditions [11].

Diagnostic yield is higher when genetic testing is directed toward individuals with an increased pre-test probability of carrying the alteration of interest. For FMR1, the literature shows that the overall yield is only 1-2% when testing is performed broadly in unselected populations, but increases substantially when restricted to individuals with a compatible family history [23]. Therefore, the higher yield observed in our study likely reflects the fact that testing was limited to cases with specific clinical indications, which naturally increases the likelihood of detecting positive results.

Some inherited metabolic disorders may present with a phenotype consistent with GDD or IDD. They are particularly relevant in children under two to three years of age who exhibit psychomotor regression or stagnation, acute encephalopathy or altered consciousness, movement disorders, treatment-resistant seizures, and specific ophthalmologic findings, among others. Patients with these red flag signs should be promptly referred to a specialist [11]. However, due to the implementation of the national newborn screening program, most of these conditions are now diagnosed early, during the neonatal period, allowing timely initiation of specific treatment, which in many cases prevents progression to GDD/IDD [8]. According to recent literature, the diagnostic yield of metabolic testing in these contexts ranges from 0.25% to 0.42% [11]. In the present study, no cases of metabolic disease were identified among the children evaluated.

Next-generation sequencing enables the simultaneous analysis of hundreds to thousands of genes from a single DNA sample [11,22]. ES (Exome Sequencing) has a high diagnostic yield, is cost-effective, and demonstrates proven clinical utility, making it strongly recommended for individuals with GDD or IDD [24]. ES is recommended as a first-line diagnostic test in several international protocols [22,25]. Nonetheless, ES is generally considered a second-line test in practice, performed after CMA, which also offers good diagnostic yield, is more widely available, and has a lower cost [26]. At our hospital, ES is currently not available.

This study has several limitations. It was conducted at a single district hospital, which may limit the generalizability of the findings. The cohort consisted predominantly of children with mild forms of GDD and IDD, which likely contributed to the relatively low diagnostic yield. In addition, some of the assessment tools used were earlier versions, which may have affected diagnostic precision. Access to advanced genetic testing, particularly exome sequencing, was not available during the study period, further limiting etiological clarification. Finally, certain investigations were performed only in selected cases, which may have led to underdiagnosis of rare conditions.

## Conclusions

This study demonstrates that a structured clinical protocol can standardize and improve the diagnostic approach to children with suspected GDD or IDD. Although the overall diagnostic yield was modest, likely reflecting the predominance of mild phenotypes and the limited availability of advanced genetic testing, first-line evaluations such as CMA, targeted karyotyping, and FMR1 analysis proved valuable when guided by clinical indications. Autism spectrum disorder emerged as the most frequent comorbidity, supporting systematic screening in children with developmental concerns. A genetic etiology was identified in 11% of cases, emphasizing both the usefulness of genetic evaluation and the challenges in achieving etiological clarification. Overall, these findings support broader access to genomic technologies, multidisciplinary assessment, and early referral to optimize outcomes for children with developmental disorders.
